# Gastroesophageal Cancer: Advances in Clinical Diagnosis and Management

**DOI:** 10.3390/jcm15218663

**Published:** 2026-11-06

**Authors:** Maximos Frountzas, Tania Triantafyllou

**Affiliations:** 1 Department of Colorectal Surgery, The Royal Marsden Hospital, NHS Foundation Trust, London SW73LD, UK; 2 1st Propaedeutic Surgical Department, National and Kapodistrian University of Athens, Hippokrateion General Hospital, 11527 Athens, Greece

## 1. Introduction

Gastroesophageal cancer remains among the most lethal malignancies worldwide, ranking highly in both incidence and cancer-related mortality [1]. Despite substantial progress in staging, systemic therapy, and surgical technique over the past two decades, outcomes continue to be shaped by late-stage presentation, treatment-related morbidity, and considerable heterogeneity in tumor biology [2]. This Special Issue, “Gastroesophageal Cancer: Advances in Clinical Diagnosis and Management,” was conceived to capture this breadth, bringing together nine contributions that span the full continuum of care—from population-level screening to long-term survivorship—for both esophageal and gastric malignancy. Read together, these papers trace a field that is maturing simultaneously on several fronts: earlier and more pragmatic detection, more biologically informed prognostication, more coordinated management of surgical complications, and a belated but welcome attention to long-term functional outcomes.

## 2. Early Detection and Risk Stratification

A recurring theme across the collected studies is the search for better tools to identify high-risk disease earlier and to stratify patients more precisely once a diagnosis is made. Xiang and colleagues addressed the persistent gap in organized screening programs by validating a two-step, questionnaire-and-serology strategy for opportunistic endoscopic screening of gastric cancer [3]. Drawing on nearly 60,000 participants in the SIGES cohort, their model identified male sex, age over 40, family history of gastric cancer, upper digestive tract benign disease, anemia, and elevated CEA or CA19-9 as independent risk factors, and demonstrated that a sequential, cost-conscious protocol could match the detection rate of more resource-intensive alternatives. This work is a timely reminder that, in the absence of nationwide screening infrastructure, pragmatic risk stratification remains one of the most cost-effective levers available to improve early detection, particularly in health systems where universal endoscopic screening is not feasible [4].

## 3. Prognostic Biomarkers and Tumor Biology

Once disease is diagnosed, prognostic stratification becomes central to treatment planning, and several papers in this Special Issue advance that effort. Yakin et al. examined 155 patients undergoing trimodality treatment for non-metastatic esophageal cancer, confirming that histology and stage retain powerful prognostic value: squamous cell carcinoma carried a substantially more favorable trajectory than adenocarcinoma, and stage IVa disease conferred an approximately fivefold increase in mortality risk [5]. Notably, their data support a role for active surveillance in carefully selected patients achieving a complete clinical response, sparing some from the perioperative risk of surgery without an apparent cost to survival.

Complementing this, Oyucu Orhan and colleagues turned to the immune and inflammatory microenvironment in gastric and gastroesophageal junction adenocarcinoma, showing that lymphovascular invasion and pan-cytokeratin positivity, alongside the monocyte-to-lymphocyte ratio, were independent predictors of survival after neoadjuvant chemotherapy—arguably more informative than the choice of regimen itself [6]. Their integration of real-world data with a broader literature synthesis reinforces a shift in thinking: tumor biology and host inflammatory response may outweigh regimen selection in determining long-term outcome, and readily available hematological indices may offer meaningful risk stratification even in settings where molecular profiling is not routinely available [7].

## 4. Adjuvant Treatment Strategy

The question of adjuvant treatment intensity in gastric cancer was tackled directly by Yildirim et al., whose retrospective, propensity-weighted comparison of adjuvant chemoradiotherapy versus chemotherapy alone found that, once baseline imbalances in tumor stage and nodal status were accounted for, the apparent survival disadvantage associated with chemoradiotherapy largely disappeared [8]. Their findings underscore both the risk of confounding by indication in observational treatment comparisons and the enduring prognostic weight of T stage, N stage, vascular invasion, and margin status—factors that recur, in one form or another, throughout this Special Issue. The study is a useful methodological counterpoint as well, illustrating how propensity-score adjustment can help disentangle true treatment effect from the selection bias inherent in real-world, non-randomized cohorts [9].

## 5. The Emerging Role of Immunotherapy in Resectable Gastric Adenocarcinoma

Beyond the biomarker- and regimen-focused work described above, the treatment landscape for resectable gastric and gastroesophageal junction adenocarcinoma is being reshaped by the incorporation of immune checkpoint inhibition into curative-intent therapy [10]. There has been a growing body of perioperative trial evidence combining immune checkpoint inhibitors with standard chemotherapy backbones, noting consistent improvements in pathological complete response rates alongside encouraging, though still maturing, event-free and overall survival signals. At the same time, there are certain remaining uncertainties in the field: the optimal duration and sequencing of perioperative immunotherapy, the reliability of PD-L1 combined positive score as a predictive biomarker outside the metastatic setting, and the need for additional, validated biomarkers to identify which patients with microsatellite-stable, non-EBV tumors are likely to benefit. These themes resonate directly with the prognostic and biomarker-focused contributions elsewhere in this Special Issue, particularly Oyucu Orhan et al.’s work on inflammatory and immunohistochemical predictors of chemotherapy response, and together they point toward a near future in which treatment selection for resectable gastric adenocarcinoma is guided as much by tumor immunobiology as by anatomical stage.

## 6. Management of Surgical Complications

Surgical and postoperative complication management is addressed from complementary angles. Riva et al. and Triantafyllou et al. each examined the management of anastomotic leak, still one of the most feared complications of esophago-gastric surgery [11,12]. The Milan series comparing self-expanding metal stents with endoscopic vacuum therapy found comparable clinical success but a markedly lower complication rate with vacuum therapy, particularly favoring its use for esophago-jejunal leaks. The Athens series, describing a six-year, multidisciplinary experience integrating surgical, radiological, and endoscopic approaches, achieved a 90-day mortality of only 3.4%—a result that highlights how far outcomes have advanced when leak management is approached as a coordinated, algorithm-driven process rather than a single-modality decision. Together, these two contributions offer complementary evidence that the shift away from reflexive reoperation, toward endoscopic-first strategies embedded within multidisciplinary pathways, is now delivering measurable gains in morbidity and mortality.

## 7. Survivorship and Quality of Life

Looking beyond the acute complication, Dellaportas and colleagues examined a less-studied consequence of esophagectomy: dumping syndrome [13]. Using validated symptom-based instruments in long-term survivors, they found that a meaningful proportion of patients experienced significant dumping-related symptoms, with measurable negative effects on dysphagia, eating restriction, and overall quality of life. As survival after esophagectomy continues to improve, this contribution is a valuable reminder that survivorship, not just survival, deserves the same systematic, instrument-based attention that has long been applied to oncological endpoints [14].

## 8. Rare Entities and Emerging Technologies

Two further contributions broaden the scope of this Special Issue. Sotiropoulou et al. presented a detailed case of oligometastatic mixed neuroendocrine-non-neuroendocrine neoplasm of the esophagogastric junction, successfully managed with induction chemotherapy targeting the aggressive neuroendocrine component followed by conversion surgery [15]. Together with their review of the sparse existing literature, this report offers practical guidance for a rare and diagnostically challenging entity for which no formal treatment guidelines currently exist [16].

Finally, Mela and colleagues provided a forward-looking narrative review of artificial intelligence applications across the esophageal cancer care pathway, from treatment planning and surgical phase recognition to response prediction and prognostication [17]. Their synthesis of more than 40 studies illustrates both the promise of these tools—several already matching or exceeding expert performance in narrow tasks—and the substantial work still needed on external validation, explainability, and integration into multidisciplinary decision-making before artificial intelligence can be considered a standard clinical tool rather than a research curiosity.

Robotic platforms represent a parallel technological advance already embedded in routine surgical practice. Reporting their experience of more than 200 robotic-assisted minimally invasive esophagectomies, Matto et al. describe how the enhanced dexterity, three-dimensional visualization, and improved ergonomics afforded by the robotic platform facilitate more meticulous periesophageal and mediastinal lymphadenectomy, with their series suggesting a higher median lymph node yield compared to conventional minimally invasive esophagectomy [18]. Given that adequate nodal harvest is directly linked to accurate pathological staging and, potentially, to oncological outcome, this technical refinement complements the biomarker- and imaging-based advances described elsewhere in this Special Issue, reinforcing the theme that improvements in staging precision are being pursued simultaneously at the molecular and the technical level [19].

## 9. Conclusions

**Figure 1:**
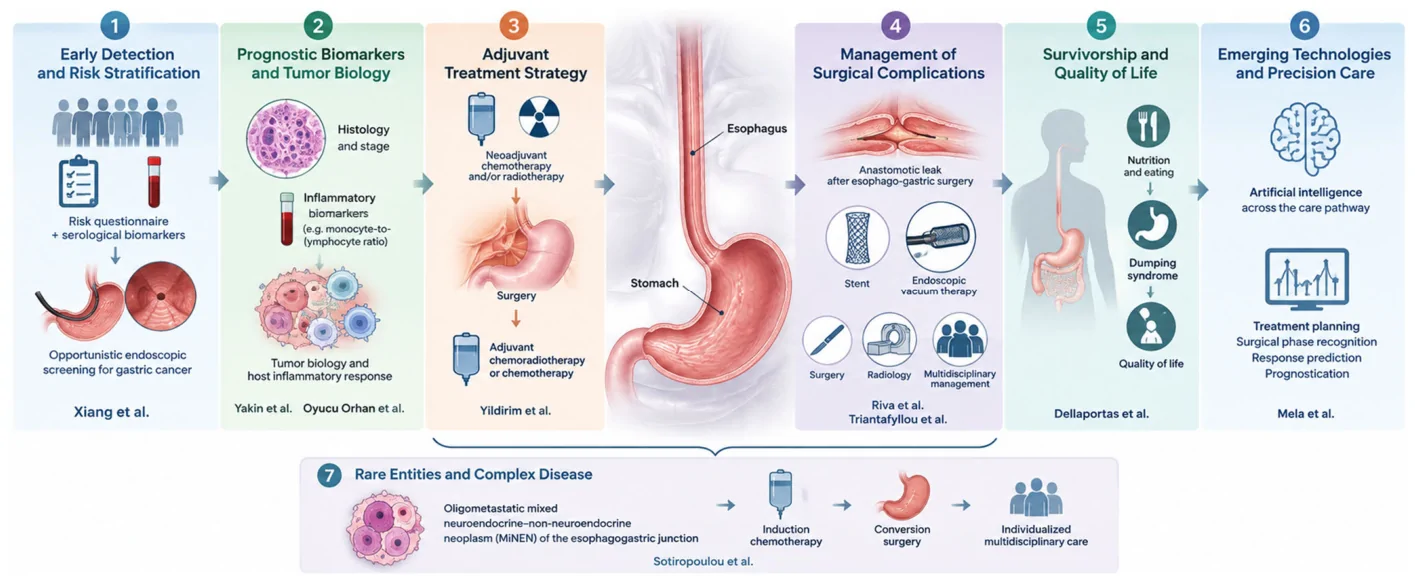
Overview of the evolving continuum of gastroesophageal cancer care, from early detection and risk stratification to multidisciplinary treatment, survivorship, and precision medicine [3,5,6,8,11,12,13,15,17].

Taken together, the contributions in this Special Issue illustrate a field that is maturing along multiple fronts simultaneously: risk-based screening is becoming more pragmatic and cost-aware; prognostic models increasingly draw on tumor biology and host inflammatory status rather than stage alone; the incorporation of immunotherapy into curative-intent treatment is beginning to redefine perioperative management for molecularly selected patients; multimodal and minimally invasive strategies for managing surgical complications are reducing morbidity and mortality; and attention to long-term functional outcomes is finally catching up with improvements in survival (Figure 1). Artificial intelligence, refinements in robotic surgical technique, and refined biomarker panels offer plausible paths toward more individualized care, though their translation into routine practice will require the same rigor—multicenter validation, transparent reporting, and attention to real-world heterogeneity—that characterizes the best work collected here. We thank all contributing authors and reviewers for their efforts, and we hope this collection serves as both a useful reference and a stimulus for the prospective, collaborative studies that this field still needs.

## Data Availability

Data available upon justified request to study authors.
